# VEGF Triggers Transient Induction of Autophagy in Endothelial Cells via AMPKα1

**DOI:** 10.3390/cells9030687

**Published:** 2020-03-11

**Authors:** Katrin Spengler, Nderim Kryeziu, Silke Große, Alexander S. Mosig, Regine Heller

**Affiliations:** 1 Institute of Molecular Cell Biology, Center for Molecular Biomedicine, Jena University Hospital, 07743 Jena, Germany; Katrin.Spengler@med.uni-jena.de (K.S.); nderim_kryeziu@hotmail.com (N.K.); Silke.Lindenmueller@med.uni-jena.de (S.G.); 2 Institute of Biochemistry II and Center for Sepsis Control and Care, Jena University Hospital, 07743 Jena, Germany; Alexander.Mosig@med.uni-jena.de

**Keywords:** AMPK, autophagy, angiogenesis, VEGF, mTOR, ULK1

## Abstract

AMP-activated protein kinase (AMPK) is activated by vascular endothelial growth factor (VEGF) in endothelial cells and it is significantly involved in VEGF-induced angiogenesis. This study investigates whether the VEGF/AMPK pathway regulates autophagy in endothelial cells and whether this is linked to its pro-angiogenic role. We show that VEGF leads to AMPKα1-dependent phosphorylation of Unc-51-like kinase 1 (ULK1) at its serine residue 556 and to the subsequent phosphorylation of the ULK1 substrate ATG14. This triggers initiation of autophagy as shown by phosphorylation of ATG16L1 and conjugation of the microtubule-associated protein light chain 3B, which indicates autophagosome formation; this is followed by increased autophagic flux measured in the presence of bafilomycin A1 and by reduced expression of the autophagy substrate p62. VEGF-induced autophagy is transient and probably terminated by mechanistic target of rapamycin (mTOR), which is activated by VEGF in a delayed manner. We show that functional autophagy is required for VEGF-induced angiogenesis and may have specific functions in addition to maintaining homeostasis. In line with this, inhibition of autophagy impaired VEGF-mediated formation of the Notch intracellular domain, a critical regulator of angiogenesis. Our study characterizes autophagy induction as a pro-angiogenic function of the VEGF/AMPK pathway and suggests that timely activation of autophagy-initiating pathways may help to initiate angiogenesis.

## 1. Introduction

Macroautophagy (herein, autophagy) is a cellular self-digestion process by which intracellular components such as long-lived proteins or dysfunctional organelles are sequestered in double-membrane autophagosomes and targeted to lysosomes for degradation [1]. While basal autophagy maintains cellular homeostasis under normal growth conditions, autophagy activated by nutrient or energy depletion serves to recycle essential biomolecules for cell survival and growth [2,3,4,5]. Autophagy stimulation is mediated via nutrient-sensing pathways involving the inhibition of mechanistic target of rapamycin (mTOR) and/or the activation of AMP-activated protein kinase (AMPK). mTOR and AMPK are serine (S)/threonine (T) kinases, which both target the Unc-51-like kinase 1 (ULK1), a key initiator of autophagy, and which also negatively regulate each other [6]. While mTOR inhibits ULK1 activity by phosphorylating its S758 residue (human sequence, corresponding to murine S757), AMPK triggers activation of ULK1 via phosphorylation of several serine sites including S556 (human sequence, corresponding to murine S555) [7,8,9,10,11], although inhibitory effects of AMPK have also been observed [12]. Once ULK1 is activated, it phosphorylates several components of the vacuolar protein sorting 34/phosphoinositide 3-kinase (VPS34/PI3K) complex leading to generation of phosphatidylinositol 3-phosphate (PI3P) and nucleation of the autophagosome [13,14]. The formation of the autophagosome is then completed by protein conjugation and lipidation complexes that involve the conversion of the microtubule-associated protein light chain 3B-I (LC3B-I) to LC3B-II [15]. The autophagosome finally fuses with a lysosome to allow degradation of the cargo by lysosomal proteases [16].

In endothelial cells, autophagy is regulated by laminar shear stress [17,18] and protects endothelial cells from oxidative stress, high glucose or excess lipid accumulation [19,20,21,22,23,24]. Several vasculo-protective compounds such as epigallocatechin gallate, resveratrol and curcumin [23,25,26] or endothelial stressors, for example, oxidized low density lipoproteins [27,28] or advanced glycation end products [29] have been shown to stimulate autophagy thereby strengthening endothelial resistance or compensating for the detrimental effects of cellular stress, respectively. Loss of autophagy has been reported to cause endothelial dysfunction in aged human and mouse endothelial cells [30,31] and to accelerate atherosclerotic plaque formation in mice [32]. Autophagy seems to be required for maintaining endothelial functions such as nitric oxide (NO) biosynthesis [33,34,35], endothelial barrier function [36,37], secretion of von Willebrand factor [38], and angiogenesis [39,40,41,42,43,44]. In contrast, an anti-angiogenic effect of autophagy induction has also been reported [45,46,47].

The induction of autophagy in endothelial cells by various agonists often involves the activation of AMPK, which suggests that autophagy is one of the mechanisms by which AMPK accomplishes its known anti-inflammatory and anti-atherogenic effects [21,25,40,47,48,49,50]. AMPK is ubiquitously expressed and mainly known as a sensor and regulator of the cellular energy status [51]. It maintains cellular ATP homeostasis by activating catabolic pathways and inhibiting anabolic pathways via phosphorylation of proteins such as acetyl-CoA carboxylase (ACC), an important regulator of fatty acid metabolism [52]. Our previous work and other studies have shown that vascular endothelial growth factor (VEGF), an important angiogenic stimulus, is a potent agonist of AMPK activation and that the catalytic isoform AMPKα1 is essentially involved in VEGF-induced angiogenesis [53,54,55]. The mechanisms underlying the pro-angiogenic role of AMPK are still poorly understood. One explanation is that AMPK inhibits glutamine:fructose-6-phosphate amidotransferase 1 (GFAT1), leading to reduced formation of O-linked β-N-acetylglucosamine-modified proteins, which normally negatively affect angiogenesis [56]. Another reason may be that AMPK activates endothelial NO synthase (eNOS) and promotes angiogenesis by enhanced NO production, although data on the interaction of AMPK with eNOS are conflicting [53,57,58,59,60,61]. In the present study, we have addressed the hypothesis that the VEGF/AMPK pathway regulates autophagy in endothelial cells and that stimulation of autophagy contributes to the pro-angiogenic function of this pathway. Our data show that the growth factor VEGF leads to transient induction of autophagy in endothelial cells via sequential activation of AMPK and mTOR pathways leading to consecutive phosphorylation of ULK1 at activating (S556) and inhibiting (S758) sites.

## 2. Materials and Methods

### 2.1. Chemicals

M199 was purchased from Lonza (Verviers, Belgium), fetal calf serum (FCS) and human serum were from Sigma (Taufkirchen, Germany) and endothelial mitogen was purchased from Biomedical Technologies Inc. (Stoughton, MA, USA). The siRNAs against AMPKα1, AMPKα2, ULK1, BECN1 and non-targeting control siRNA were SMARTpool-siRNAs obtained from GE Healthcare, Dharmacon RNAi and Gene Expression (Lafayette, CO, USA). Recombinant human VEGF-165 was obtained from R&D Systems GmbH (Wiesbaden, Germany). Protease inhibitor mixture complete, EDTA-free, was obtained from Roche Diagnostics (Mannheim, Germany) and used in protein lysates, while a protease inhibitor cocktail obtained from Sigma (Taufkirchen, Germany) was administered to protect cytokine degradation in supernatants. Propidium iodide, RNase A, DL-buthionine-(S,R)-sulfoximine (BSO), 5,5′-Dithiobis(2-nitrobenzoic acid) (DTNB), NADPH, 2-deoxyglucose (2-DG), thrombin and aprotinin were purchased from Sigma (Taufkirchen, Germany). Bafilomycin A1 was from Enzo Life Sciences (Lörrach, Germany) and fibrinogen was from Merck/Millipore (Darmstadt, Germany). Bovine serum albumin-C (BSA-C) and goat serum were obtained from Aurion (Wageningen, The Netherlands) and Cell Signaling Technology (Frankfurt, Germany), respectively.

### 2.2. Antibodies

Rabbit monoclonal antibodies against β-actin, p62, BECN1, p-ULK1 (S555* (mouse), S556 (human)), p-ATG14 (S29), ATG14, ATG16L1, p-AMPKα (T172), p-VEGFR2 (Y1175), VEGFR2, p-eNOS (S1177), ACC, cleaved Notch1 (V1744) (NICD), rabbit polyclonal antibodies against p-ULK1, (S757* (mouse), S758 (human), ULK1, AMPKα, AMPKα1, AMPKα2, p-PLCγ (Y783), PLCγ, p-p70S6K (T389), p70S6K, p-ACC (S79) and mouse monoclonal antibodies against LC3B, p-ERK1/2 (T202/Y204) and ERK1/2 were obtained from Cell Signaling Technology (Frankfurt, Germany). The antibodies labelled with * are named after the murine sequences by the manufacturer but also recognize the respective human sequences. The LC3B antibody detected mainly the type II form of LC3B under the applied conditions. Rabbit monoclonal antibody against p-ATG16L1 (S278) and mouse polyclonal antibody against eNOS were from Abcam (Cambridge, UK) and BD Transduction Laboratories (Heidelberg, Germany), respectively. Peroxidase-labeled anti-mouse and anti-rabbit IgG were from Kirkegaard and Perry Laboratories, Inc. (Gaithersburg, MD, USA). Secondary AlexaFluor^®^488-conjugated goat anti-rabbit IgG was from Thermo Scientific (Waltham, MA, USA).

### 2.3. Cell Culture

Human umbilical vein endothelial cells (HUVEC) were isolated from anonymously acquired umbilical cords according to the Declaration of Helsinki, “Ethical principles for Medical Research Involving Human Subjects” (1964). The study was approved by the Jena University Hospital Ethics Committee (no. 3130-05/11) and donors were informed and gave written consent. For cell preparation, umbilical cord veins were cleaned with 0.9% NaCl solution and cells were detached with 0.01% collagenase dissolved in M199 for 3 min at 37 °C. Veins were then rinsed with M199/10% FCS and the cell suspension was centrifuged (500× g, 6 min). The pellet was resuspended in M199/10% FCS and seeded on a cell culture flask coated with 0.2% gelatin. After 24 h, cells were washed and cultured in full growth medium (M199, 17.5% FCS, 2.5% human serum, 7.5 µg/mL endothelial mitogen, 7.5 U/mL heparin, 680 µM glutamine, 100 µM vitamin C, 100 U/mL penicillin, 100 µg/mL streptomycin). In general, HUVEC from the second passage were seeded at a density of 27,500/cm^2^ and used for experiments three days after seeding if not otherwise indicated. For transfection with siRNA, the seeding density was 23,000/cm^2^, for cell cycle analysis 15,000/cm^2^ if cell proliferation was investigated and 23,000/cm^2^ if survival was analyzed. For the immunofluorescence studies, 50,000 cells/cm^2^ were seeded on coverslips. Most experiments were performed with cells on 30 mm culture dishes. For spheroid generation, ATP measurements or glutathione (GSH) determination, 96-well plates, 24-well plates or 60 mm dishes were employed, respectively.

### 2.4. Cell Treatment

HUVEC were incubated in full growth medium for monitoring basal autophagy and the effect of autophagy inhibition on basal cell functions (growth, survival, cytokines, GSH, ATP, validation of downregulation, mRNA expression). Experiments, in which VEGF was used as a stimulus, were either performed in the absence of serum (short-term studies, <30 min) or in the presence of 2% FCS (long-term studies, >30 min). Stimulation in serum-free medium was done in M199 containing 0.25% human serum albumin (HSA) (signaling studies shown in Figures 2 and 3) or in Hepes buffer (10 mM Hepes (pH 7.4), 145 mM NaCl, 5 mM KCl, 1 mM MgSO4, 1.5 mM CaCl2, 10 mM glucose) supplemented with 0.25% HSA (signaling studies shown in Figure 5).

### 2.5. siRNA Transfection

HUVEC were transfected 24 h after seeding with 0.5 µg/mL of non-targeting or specific siRNA using SAINT-sRNA transfection reagent (Synvolux Therapeutics B.V., Groningen, The Netherlands). If two proteins, i.e., ULK1 and BECN1, were downregulated at the same time by using 0.5 µg/mL specific siRNA for each target, 1 µg/mL control siRNA was applied. The transfection solution was prepared by first combining siRNA diluted in 100 µL Hepes-buffered saline (HBS) with 20 µL Saint-sRNA diluted in 80 µL HBS and then adding 800 µL of M199 supplemented with 0.25% HSA to this mixture. HUVEC were washed twice with Hanks’ Balanced Salt Solution (HBSS) and the transfection solution was added. After 4 h, 2 mL of full growth medium was added and cells were cultured for 72 h before the experiment was performed.

### 2.6. Cell Lysis and Western Blot

HUVEC were lysed in ice-cold Tris buffer (50 mM Tris (pH 7.4), 2 mM EDTA, 1 mM EGTA, 50 mM NaF, 10 mM Na_4_P_2_O_7_, 1 mM Na_3_VO_4_, 1 mM DTT, 1% Triton X-100, 0.1% SDS, 1 mM PMSF, 10 µL/mL protease inhibitor cocktail (Roche) for 15 min on ice and scraped. After centrifugation of the lysates (700× *g*, 6 min), the protein content of the supernatants was determined using Lowry reagents (DC^TM^ Protein Assay kit) and bovine serum albumin (BSA) as standard. Supernatants were supplemented with Laemmli buffer, subjected to SDS-PAGE (25–50 µg lysate protein/lane) and transferred onto polyvinylidene fluoride (PVDF) membranes. The membranes were blocked for 1 h in Tris-buffered saline/Tween (TBST) buffer (20 mM Tris (pH 7.6), 137 mM NaCl, 0.1% (v/v) Tween^®^ 20) containing 5% non-fat dried skimmed milk and then incubated overnight at 4 °C with primary antibodies. Antibody dilutions were prepared in TBST containing 5% BSA. Following incubation with the respective horseradish peroxidase-conjugated secondary antibodies for 1 h, signal detection was performed using the enhanced chemiluminescence (ECL) reagent (GE Healthcare, Chicago, IL, USA) or Western Lightning Plus-ECL reagent (Perkin Elmer, Waltham, MA, US). For each staining, dilution series of lysates, antibody dilution and exposure times were tested prior to experiments and conditions were chosen that allowed protein band detection in the linear range. Quantification was carried out by densitometry using ImageJ software. If applicable, ratios between phosphorylated protein and total protein were calculated. LC3B conjugation was evaluated by analyzing LC3B type II, i.e., the lower LC3B-positive band if double bands were seen.

### 2.7. Cytokine Measurement

Experiments were started 48 h after transfection of cells with siRNA against ULK1 and BECN1 by adding 600 µL fresh growth medium and an additional incubation for 24 h. Supernatants were harvested and supplemented with protease inhibitor cocktail (Sigma, 1:500). Cytokines were measured using the BD Cytometric Bead Array (CBA) Human Soluble Protein Master Buffer Kit for IL-8 and the BD CBA Human Enhanced Sensitivity Master Buffer Kit for IL-1β and TNF-α, respectively (BD Biosciences, Heidelberg, Germany). For normalization, parallel dishes were lysed with solubilization buffer (100 mM NaOH, 1.9 M Na_2_CO_3_, 1% SDS) and protein content was determined according to Lowry.

### 2.8. GSH Measurement

After transfection of cells with siRNA to downregulate autophagy, cells were washed twice with phosphate-buffered saline (PBS) and lysed with 90 µL of 0.01 N HCl. Cells were harvested and centrifuged (15,000× g, 10 min). Sulfosalicylic acid (27.2 µL) was added to 80 µL of the supernatants and incubated on ice for 5 min. Thereafter, proteins were precipitated by centrifugation (15,000× g, 5 min). Supernatant (40 µL) was mixed with 340 µL reaction buffer (200 mM Tris, 1 mM EDTA, 1.5 mM NADPH) and 20 µL of 10 mM DTNB. The assay mixture was transferred to 96-well plates (100 µL/well, triplicates) and 20 µL glutathione reductase (10 U/mL) was added per well. The absorption at 412 nm was measured over a 12 min time period and GSH concentrations were determined on the basis of a calibration curve. Cells were lysed with solubilization buffer and protein content was determined according to Lowry for normalization.

### 2.9. Cell Cycle Analysis

Cells were trypsinized and pooled with cells from culture supernatants and washing solutions if subG_1_ fractions were analyzed. The cell suspensions were centrifuged (500× g, 6 min) and the obtained cell pellets washed twice with PBS and resuspended in 200 µL cold PBS. Then, 2 mL 70% ethanol was added dropwise and mixed carefully and samples were incubated for 1 h at 4 °C under gentle shaking. Pellets were washed twice in cold PBS with 0.5% Tween^®^ 20, resuspended in 100 µL RNase A solution (200 µg/mL) and incubated at 37 °C for 15 min. Then, 100 µL propidium iodide (150 µg/mL) was added and incubated for 10 min in the dark. Samples were then analyzed by flow cytometry.

### 2.10. Mitochondrial ROS Measurement

Mitochondrial ROS levels were detected using the MitoSOX™ Red Mitochondrial Superoxide Indicator from Thermo Scientific (Waltham, MA, USA). Experiments were performed according to the manufacturer’s protocol. In brief, cells were washed once with PBS and incubated with staining solution for 30 min. After trypsinization cell pellets were resuspended in PBS and subjected to flow cytometric analysis.

### 2.11. ATP Measurement

After transfection with siRNA against ULK1 and BECN1 and additional experimental incubations, cell proteins were denaturated by adding 200 µL ethanol per well. After evaporation of the ethanol, 100 µL Tris buffer contained in the assay kit was added and cells were subjected to one freezing/thawing cycle in liquid nitrogen, scraping and centrifugation. The ATP content of the soluble fraction was determined using the ATP Kit SL from Biotherma (Handen, Sweden) according to the manufacturer’s protocol. For normalization, cells in identically treated wells were lysed with solubilization buffer and the protein content was determined according to Lowry.

### 2.12. Spheroid Assay

Spheroids were generated as described previously [62]. In brief, control or siRNA-transfected cells suspended in growth medium were mixed with methyl cellulose (stock solution 12 mg/mL) at a 4:1 ratio and 3000 cells/well were seeded in 96-well round-bottom plates. After 24 h, spheroids were collected, centrifuged (200× g, 4 min) and washed with Hepes buffer. Then, fibrinogen solution (1.8 mg/mL in Hepes buffer) containing 20 U/mL aprotinin was added to the spheroids to obtain a suspension with approximately 100 spheroids per ml. Then, 300 µL of this suspension together with 0.2 U thrombin were added per well of a 24-well plate. The plate was incubated for 20 min at 37 °C to allow the formation of a fibrin gel. To equilibrate the gel with medium and wash out thrombin, M199 containing 2% FCS, 680 µM glutamine, 100 U/mL penicillin and 100 µg/mL streptomycin was added twice for 15 min. Thereafter, spheroids were cultured in the same medium and stimulated with 50 ng/mL VEGF for 24–48 h. Finally, spheroids were fixed on ice by adding 1 mL 4% paraformaldehyde per well for 10 min. After two washing steps with PBS, spheroid sprouting was viewed by light microscopy and pictures were taken (AxioVert 200, Carl Zeiss, Oberkochen, Germany). The number and length of sprouts were analyzed using cellSens image analysis software (Olympus, Tokyo, Japan).

### 2.13. qRT-PCR

RNA was extracted using the NucleoSpin^®^ Kit from Machery-Nagel (Düren, Germany). cDNA from 1500 ng RNA was synthesized with the First Strand cDNA Synthesis Kit from Thermo Scientific (Waltham, MA, USA). Quantitative RT-PCR was performed with the Maxima SYBR Green/ROX qPCR Master Mix (2X) from Thermo Scientific using primers for LC3B (forward 5′GATGTCCGACTTATTCGAGAGC3′′, reverse 5′TTGAGCTGTAAGCGCCTTCTA3′) and β-actin (forward 5′GGGACGACATGGAGAAAATCTG3′, reverse 5′GAAGGTCTCAAACATGATCTGGG3′). LC3B expression was normalized to the expression of β-actin.

### 2.14. LC3B Immunofluorescence

Seventy-two hours after transfection with siRNA, cells were washed with Hepes buffer and fixed with 500 µL ice-cold methanol for 10 min at 4 °C. After two washes with ice-cold PBS 300 µL blocking solution (10% BSA-C, 5% goat serum in PBS) was added and coverslips were incubated for 30 min at room temperature. Cells were washed twice with PBS followed by incubation with 50 µL of LC3B antibody solution (1:200 in blocking solution) for 2 h. Subsequently, cells were washed twice with PBS, incubated with 50 µL AF488-conjugated secondary antibody (1:500 in blocking solution) for 1 h and mounted on slides. Pictures were taken with the Olympus BX61 microscope (Olympus, Tokyo, Japan).

### 2.15. Statistics

Data are given as mean values ± SEM from at least three independent experiments. For testing statistical significance one-way or two-way repeated measurement ANOVA with Holm–Šidák post hoc testing were used to compare two or more conditions, respectively. A p-value of <0.05 was accepted as statistically significant.

## 3. Results

### 3.1. Functional Autophagy is a Prerequisite for Endothelial Homeostasis

**Figure 1:**
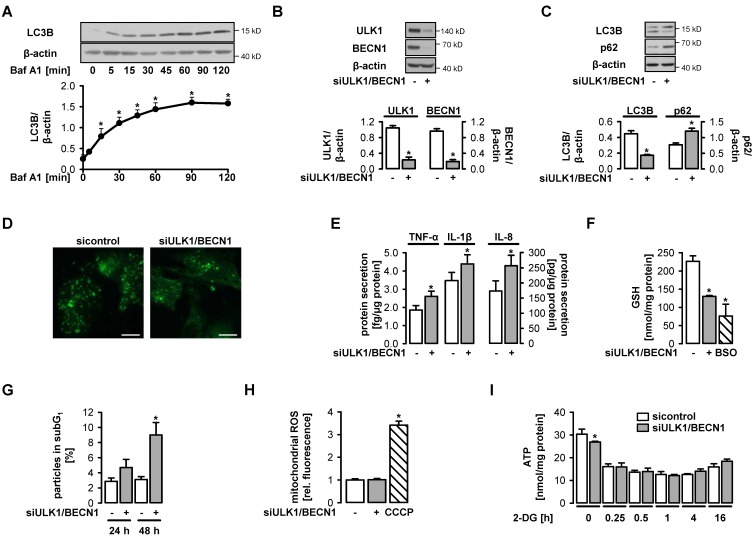
Functional autophagy is a prerequisite for endothelial homeostasis. (**A**) Human umbilical vein endothelial cells (HUVEC) were treated with 50 nM bafilomycin A1 (Baf A1) for the indicated times. Lysates were subjected to Western blot analyses of LC3B and β-actin. Representative blots and densitometric evaluation are shown (mean values + SEM, n = 5), * *p* < 0.05 vs. untreated control. (**B**–**I**) HUVEC were transfected with control-siRNA or Unc-51-like kinase 1 (ULK1)- plus beclin 1 (BECN1)-siRNA for 72 h and analyzed thereafter (**B**–**F**, **H**–**I**) or after cultivation for 24 or 48 h (**G**). (**B**–**C**) Cell lysates were analyzed for the indicated proteins in Western blots. Representative blots and densitometric evaluation are shown (mean values + SEM, n = 5). (**D**) Cells were stained for LC3B, whose accumulation in punctae reflects the formation of autophagosomes. Representative immunofluorescent images are shown (n = 2), scale bar = 10 µm. (**E**) Cytokines were quantified in cell supernatants by multiplex bead-based flow cytometric analyses (mean values + SEM, n = 5). (**F**) Glutathione (GSH) levels of cell lysates were determined in a colorimetric assay (mean values + SEM, n = 3). The positive control was treated with 100 µM DL-buthionine-(S,R)-sulfoximine (BSO, inhibitor of GSH synthesis) for 12 h. (**G**) Cells were stained with propidium iodide and analyzed by flow cytometry. The percentage of particles in the subG_1_ fraction is shown (mean values + SEM, n = 5). (**H**) Mitochondrial production of reactive oxygen species was detected by MitoSOX-based flow cytometry. Treatment of cells with 100 µM carbonyl cyanide *m*-chlorophenyl hydrazone (CCCP) for 1 h served as positive control. (**I**) Cells were treated with 20 mM 2-deoxyglucose (2-DG) for the indicated times. ATP levels in cell extracts were measured using a luciferase-based assay (mean values + SEM, n = 3). Compared to untreated controls, 2-DG led to a significant reduction of ATP levels under all conditions (*p* < 0.001, not indicated in the graph). (**B**–**I**) * *p* < 0.05 vs. control-siRNA-treated cells.

In the first series of experiments, we characterized autophagy in human umbilical vein endothelial cells cultured in full growth medium. These cells exhibited substantial basal autophagic flux, which was shown as time-dependent accumulation of conjugated LC3B in the presence of the lysosomal inhibitor bafilomycin A1 (Figure 1A). To study the role of autophagy, ULK1 and beclin 1 (BECN1), two molecules involved in autophagy initiation, were downregulated by specific siRNA (Figure 1B). This led to a decrease in LC3B conjugation, a decline in LC3B-positive autophagosomes and an accumulation of p62, a well-known autophagy substrate, indicating a significant reduction in autophagy in comparison to control cells (Figure 1C,D). Autophagy inhibition was paralleled by enhanced secretion of the pro-inflammatory cytokines, tumor necrosis factor-α (TNF-α), interleukin-1β (IL-1β) and interleukin-8 (IL-8), decreased levels of the endogenous antioxidant glutathione (GSH) suggesting impaired antioxidant defense, and impaired cell survival (Figure 1E–G). Mitochondrial formation of reactive oxygen species (ROS) was not enhanced under these conditions (Figure 1H). Downregulation of ULK1 and BECN1 had little effect on ATP availability. When endothelial cells were additionally challenged with the glycolysis inhibitor 2-deoxyglucose (2-DG), which led to ATP reduction, no difference in ATP levels between control cells and autophagy-deficient cells was seen (Figure 1I). Together, these data underline the important homeostatic role of basal autophagy in endothelial cells but do not suggest that this process has an influence on energy metabolism.

### 3.2. VEGF Initiates Functional Autophagy in Endothelial Cells via Phosphorylation of ULK1 at S556

**Figure 2:**
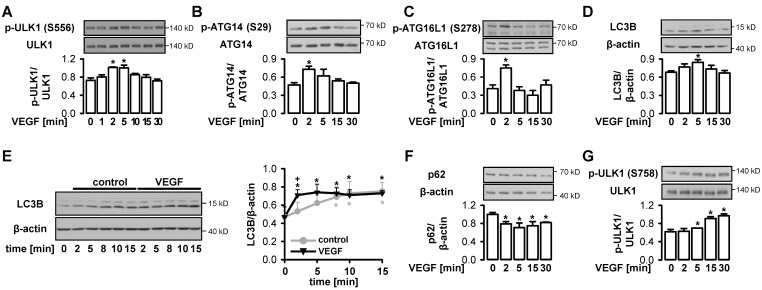
Vascular endothelial growth factor (VEGF), a physiological AMP-activated protein kinase (AMPK) agonist, initiates autophagy in endothelial cells via phosphorylation of ULK1 at S556. (**A**–**D**, **F**–**G**) HUVEC were stimulated with 50 ng/mL VEGF for the indicated times, lysed and subjected to Western blot analyses of the indicated proteins. (**E**) HUVEC were pretreated with 50 nM bafilomycin A1 for 15 min (time = 0), subsequently stimulated with 50 ng/mL VEGF or vehicle (control) for the indicated times, lysed and subjected to Western blot analyses of LC3B. (**A**–**G**) Representative blots and densitometric evaluation are shown (mean values + SEM, n = 3 (**B**,**G**), n = 4 (**A**, **C**, **D**, **F**), n = 5 (**E**)), * *p* < 0.05 vs. unstimulated control, + *p* < 0.05 vs. unstimulated control pretreated with bafilomycin A1.

Since autophagy is known to be controlled by AMPK and mTOR, we asked how the growth factor VEGF, known to activate both pathways [53,55,63] affects autophagy. Figure 2A,B show that VEGF triggered transient phosphorylation of ULK1 and its substrate ATG14, a member of the VPS34 complex [64], at S556 and S29, respectively, denoting the initiation of autophagy. Accordingly, a transitory phosphorylation of ATG16L1, a part of the LC3B lipidation complex [65,66], at S278 and conjugation of LC3B, which both point to the formation of autophagosomes in response to VEGF, were observed (Figure 2C,D). Further, an early increase in autophagic flux in cells stimulated with VEGF in the presence of bafilomycin A1, and in parallel, a lower expression of p62 upon VEGF treatment were detected, indicating functional autophagy (Figure 2E,F). The transient nature of autophagy induction may be related to the inhibitory phosphorylation of ULK1 at S758, the mTOR phosphorylation site, which occurred with a time lag in response to VEGF and may terminate activation of ULK1 (Figure 2G). Together, these data show that VEGF induced autophagy in endothelial cells via ULK1 phosphorylation at S556.

### 3.3. VEGF-Induced Initiation of Autophagy depends on AMPKα1

**Figure 3:**
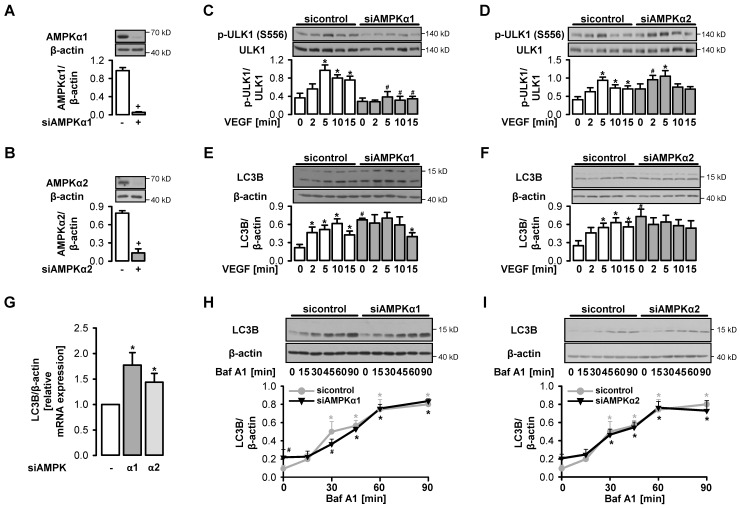
VEGF-induced ULK1 phosphorylation is mediated by AMPKα1. (**A**–**I**) HUVEC were transfected with control-siRNA, AMPKα1-siRNA or AMPKα2-siRNA, respectively. (**A**–**F**) Cells were directly lysed (**A**–**B**) or stimulated with 50 ng/mL VEGF for the indicated times (**C**–**F**) and subjected to Western blot analyses of the indicated proteins. Representative blots and densitometric evaluation are shown (mean values + SEM, n = 3 (**C**), n = 4 (**A**,**B**,**D**), n = 5 (**E**–**F**)), + *p* < 0.05 vs. control-siRNA, * *p* < 0.05 vs. unstimulated control, # *p* < 0.05 vs. the respective VEGF-stimulated sample transfected with control-siRNA. (**G**) RNA was extracted, transcribed into cDNA and qRT-PCR was performed. Mean values + SEM of LC3B mRNA levels normalized to β-actin are shown, n = 4, * *p* < 0.05 vs. control-siRNA. (**H**–**I**) Cells were treated with 50 nM bafilomycin A1 (Baf A1) for the indicated times, lysed and subjected to Western blot analyses of LC3B. Representative blots and densitometric evaluation are shown (mean values + SEM, n = 3), * *p* < 0.05 vs. respective controls without Baf A1 treatment, # *p* < 0.05 vs. respective control-siRNA-treated sample.

To understand the role of AMPK in autophagy initiation by VEGF, we applied specific siRNAs to downregulate the AMPK isoforms α1 or α2 (94% or 81% reduction, respectively) (Figure 3A,B) and compared VEGF-triggered ULK1 phosphorylation between AMPKα1- or α2-depleted and control cells. Phosphorylation of ULK1 at S556 was completely prevented when the AMPK isoform α1 was downregulated, demonstrating that it was mediated by AMPKα1 (Figure 3C). Thus, VEGF led to a transient stimulation of autophagy via activation of AMPKα1. In contrast, depletion of AMPKα2 had no effect (Figure 3D). VEGF did not induce LC3B conjugation in cells, in which AMPKα1 or AMPKα2 were downregulated (Figure 3E,F). However, under these conditions, we observed an increased basal expression and conjugation of LC3B compared to control cells as reported previously [67,68] (Figure 3E–G). This was not accompanied by an alteration of the basal autophagic flux in AMPKα1- or AMPKα2-depleted cells (Figure 3H,I).

### 3.4. VEGF-Induced Angiogenesis Requires Functional Autophagy

**Figure 4:**
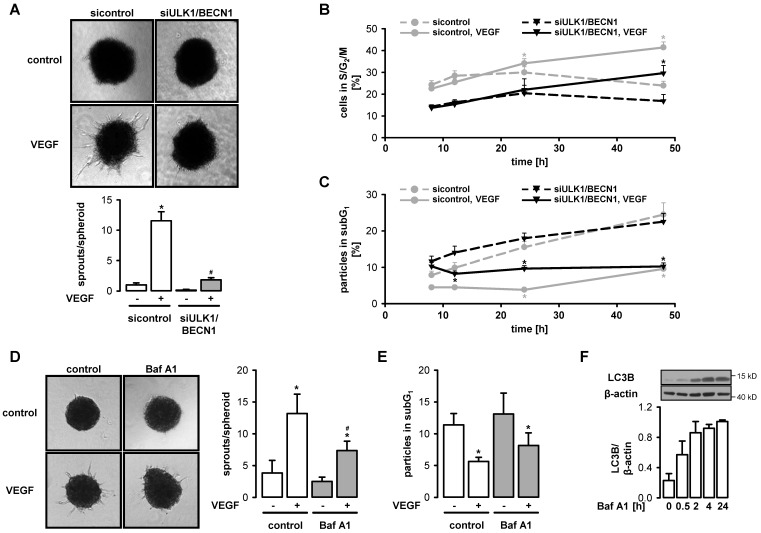
VEGF-induced angiogenesis requires functional autophagy. (**A**–**C**) HUVEC were transfected with control-siRNA or ULK1- plus BECN1-siRNA for 72 h. (**A**) Spheroids generated from transfected cells were stimulated with VEGF (50 ng/mL, 48 h) and sprouting was analyzed. Representative pictures and quantification of sprout numbers per spheroid are shown (mean values + SEM, n = 4), * *p* < 0.05 vs. respective unstimulated control, # *p* < 0.05 vs. VEGF-stimulated sample transfected with control-siRNA. (**B**–**C**) Cells were stimulated with 50 ng/mL VEGF for the indicated times and subjected to cell cycle analysis. The proportion of cells in the proliferative phases S/G_2_/M (**B**) or particles in the subG_1_ fraction (**C**) is shown (mean values + SEM, n = 4), * *p* < 0.05 vs. respective values at 8 h after VEGF addition. (**D**–**E**) HUVEC spheroids (**D**) or cultured HUVEC (**E**) were pretreated with 5 nM bafilomycin A1 (Baf A1) for 30 min and stimulated with 50 ng/mL VEGF for 24 h. (**D**) Representative pictures and the quantification of sprouts per spheroid are shown (mean values + SEM, n = 5). (**E**) Cells were stained with propidium iodide and subjected to flow cytometry analysis. The proportion of particles in the subG_1_ fraction is shown (mean values + SEM, n = 5). (**D**–**E**) * *p* < 0.05 vs. respective unstimulated control, # *p* <0.05 vs. respective non-Baf A1-treated sample. (**F**) Cells were treated with 5 nM Baf A1 for the indicated times, lysed and subjected to Western blot analysis. Representative blots and densitometric evaluation are shown (mean values + SEM, n = 2).

We next addressed the question of whether autophagy is required for VEGF-induced angiogenesis by employing a spheroid assay. VEGF induced considerable sprouting in control spheroids but had little effect in spheroids generated from ULK1/BECN1-depleted cells (Figure 4A). In contrast, VEGF was still able to stimulate proliferation and survival in autophagy-deficient cells, although basal proliferation and survival were substantially impaired, probably due to disturbed homeostasis (Figure 4B,C). Angiogenesis was also repressed by the lysosomal inhibitor bafilomycin A1 at a low concentration, which impaired autophagy but did not affect basal survival (Figure 4D–F).

### 3.5. Autophagy Interferes with VEGF-Induced Notch Signaling

**Figure 5:**
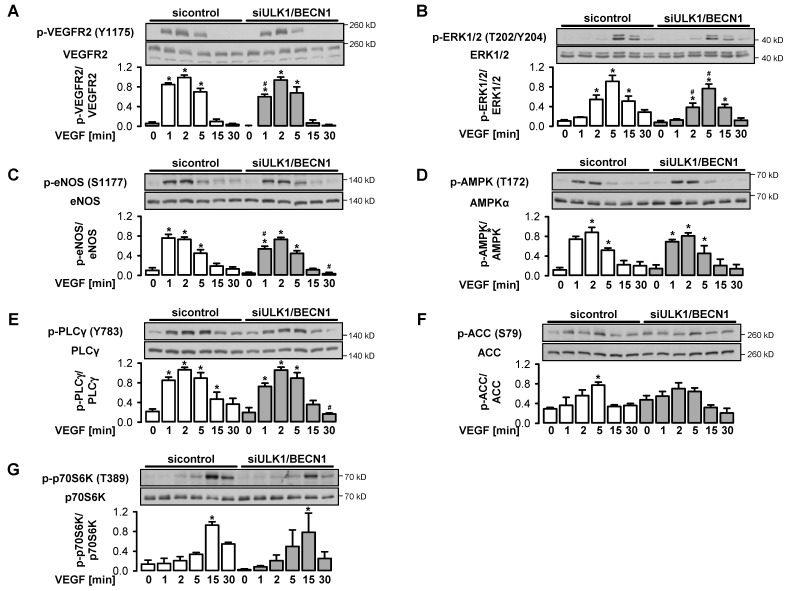
Autophagy has a minor impact on VEGF-induced signaling pathways. (**A**–**G**) HUVEC were transfected with control-siRNA or ULK1- plus BECN1-siRNA for 72 h and stimulated with 50 ng/mL VEGF for the indicated times. Cell lysates were subjected to Western blot analyses of the indicated proteins. Representative blots and densitometric evaluations are shown (mean values + SEM, n = 4), * *p* < 0.05 vs. respective unstimulated control, # *p* < 0.05 vs. respective samples treated with control-siRNA.

To test the effect of autophagy on major angiogenic signaling pathways such as activation of VEGF receptor 2 (VEGFR2), phospholipase Cγ (PLCγ), extracellular signal-regulated kinase 1/2 (ERK1/2), eNOS, p70 ribosomal protein S6 kinase (p70S6K), AMPK and ACC, control cells and ULK1/BECN1-depleted cells were stimulated with VEGF. VEGF led to a transient phosphorylation with a maximum at 2–5 min for most of the investigated proteins (Figure 5A–F). In contrast, the phosphorylation of the mTOR target p70S6K only peaked at 15 min indicating delayed activation of the mTOR pathway (Figure 5G). Almost no differences in the expression and VEGF-induced phosphorylation of the investigated proteins were seen between control cells and autophagy-deficient cells, which indicated that the respective signaling pathways were not controlled by autophagy.

**Figure 6:**
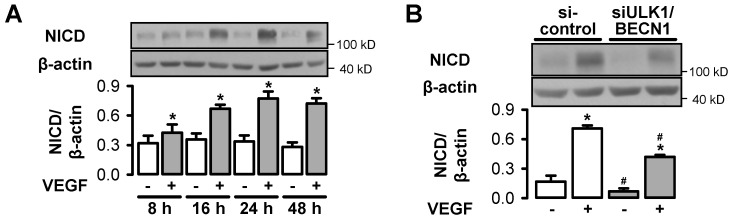
Autophagy interferes with the Notch signaling pathway. (**A**) HUVEC were stimulated with 50 ng/mL VEGF for the indicated times. (**A**) Cells were transfected with control-siRNA or ULK1- plus BECN1-siRNA and stimulated with 50 ng/mL VEGF for 24 h. (**A**–**B**) Cells were lysed and subjected to Western blot analyses of the Notch intracellular domain (NICD). Representative blots and densitometric evaluation are shown (mean values + SEM, n = 5 (**A**), n = 4 (**B**)), * *p* < 0.05 vs. respective unstimulated control, # *p* < 0.05 vs. respective samples treated with control-siRNA.

We also evaluated VEGF-induced Notch signaling, a pathway that controls endothelial differentiation and stabilization during angiogenesis, by monitoring the formation of the Notch intracellular domain (NICD). NICD accumulated time-dependently in response to VEGF in control cells and reached a maximum at 24 h (Figure 6A). At this time point, NICD abundance was significantly lower in cells in which ULK1 and BECN1 were downregulated (Figure 6B). Together, these data suggest that the failure in VEGF-induced sprout formation in conditions where autophagy is inhibited may not be related to reduced VEGF responsiveness of endothelial cells in general, but rather it may be related to disturbed basal homeostasis and to interference with specific angiogenic processes such as Notch processing.

## 4. Discussion

Autophagy is induced by inhibition of mTOR upon amino acid depletion or activation of AMPK upon glucose starvation. These two nutrient-sensing pathways converge on ULK1, a key initiator of autophagy, and regulate ULK1 activity by phosphorylating serine residues that either inhibit or activate the enzyme [6]. Growth factors such as VEGF are known to trigger the Akt/mTOR pathway [69], which mediates the inhibitory phosphorylation of ULK1, suggesting that they slow down autophagy. In line with this, growth factor removal has been reported to activate autophagy [70]. However, as shown previously by our group and others, VEGF is also a potent stimulus for AMPK activation [53,55], which in turn may lead to ULK1 activation. Thus, the question arose of how the angiogenic factor VEGF, which is activating both AMPK and mTOR pathways, affects autophagy.

Here, we report for the first time that VEGF stimulates autophagy via an AMPKα1-dependent mechanism. Autophagy activation was verified by VEGF-induced phosphorylation of ULK1 and its substrate ATG14 at sites known to be involved in the initiation of autophagy (S556 and S29, respectively) [7,8,9,10,11,64], by phosphorylation of ATG16L1, a component of the LC3B lipidation complex [65,66] and by conjugation of LC3B, a marker of autophagosome formation. Furthermore, functional autophagy was confirmed by an increase in autophagic flux soon after VEGF stimulation and a decrease in p62 expression, which indicates its enhanced autophagic degradation in response to VEGF. ULK1 phosphorylation triggered by VEGF was prevented when AMPKα1 was downregulated by specific siRNA, while downregulation of AMPKα2 had no effect. These data underline the role of AMPKα1 in VEGF-induced initiation of autophagy. In line with this, VEGF did not stimulate LC3B conjugation in AMPKα1-depleted cells, although the upregulation of LC3B in cells in which AMPKα isoforms were downregulated, hampers the interpretation of these data.

The autophagy induction in response to VEGF was transient. This may be related to mTOR having a counteracting role, which was stimulated by VEGF in a delayed manner. AMPK activity upon VEGF stimulation returned to basal levels at the same time as mTOR activity increased, which was monitored as phosphorylation of its target p70S6K [71]. In line with this, an inhibitory phosphorylation of ULK1 at S758 known to be mediated by mTOR occurred with a time delay upon VEGF stimulation and may be involved in terminating VEGF-initiated autophagy induction. In fact, mTOR-mediated phosphorylation of ULK1 at S757 (murine sequence) has previously been found to block autophagy by disrupting the interaction of AMPK with ULK1, thereby preventing ULK1 phosphorylation at S555 (murine sequence) by AMPK [7]. Thus, the sequential activation of AMPK and mTOR by VEGF may allow transient activation of autophagy, which is necessary for the induction of angiogenesis before growth-promoting pathways are initiated.

The present study reveals that induction of autophagy may be part of the pro-angiogenic processes induced by the VEGF/AMPK pathway. One function of enhanced autophagy in response to VEGF may be to create permissive conditions for the initiation of angiogenic sprouting by proteolytic clearance of damaged proteins. It is known that only fully functional endothelial cells are able to start sprouting and forming new blood vessels, while endothelial dysfunction impairs the angiogenic response [72,73]. In line with previous studies [19,31,33,34,35,36,37] our data confirm that autophagy is important to maintain endothelial functions, although it did not affect cellular energy state and mitochondrial ROS production. Autophagy blockade results in inflammatory stress, reduced antioxidative defense, reduced growth, lower survival, and finally, reduced angiogenic capacity. Conversely, stimulation of angiogenesis by chemerin, the angiogenic factor AGGF1 or by hypoxia has previously been reported to promote angiogenesis [40,43,44]. Thus, induction of autophagy may stabilize cellular homeostasis, which is required for angiogenic responses.

In addition, VEGF-induced stimulation of autophagy is likely to affect angiogenesis beyond maintaining homeostasis. It may readily provide building blocks for macromolecule synthesis necessary for sprouting. Activation of autophagy may also be required to process angiogenic molecules and support angiogenic pathways. It has already been reported that autophagy leads to degradation of angiogenic molecules like VEGFR2 and gastrin-releasing peptide (GRP) and interferes with the Wnt pathway [74,75,76], although these processes affected angiogenesis in a negative manner. In our study, VEGF-induced signaling pathways such as activation of VEGFR2, PLCγ, ERK1/2, eNOS, p70S6K and AMPK as well as the expression of these proteins were comparable in autophagy-deficient and control cells, and thus probably not regulated by autophagy. Cells with inhibited autophagy were still able to proliferate and to respond to the survival signals of VEGF to the same extent as control cells, although they started from lower basal levels. However, VEGF-induced sprouting was almost completely blocked in autophagy-deficient cells, which points to the regulatory role of autophagy in the differentiation of endothelial cells to capillary-like sprouts.

The Notch pathway is essential for regulating endothelial differentiation during angiogenesis. It is activated in cells adjacent to VEGF-activated cells leading to the release of NICD, which in turn mediates reduced responsiveness of cells to VEGF [77]. Via this pathway, tip/stalk cell specification and junctional rearrangement during angiogenesis as well as the switch from cell proliferation to vessel maturation and stabilization are controlled. Notch signaling requires tight regulation since its inhibition results in uncontrolled tip cell formation and impaired maturation, while its overstimulation leads to the breakdown of VEGFR2-mediated signaling. In stem cells, autophagy has been reported to prevent hyperactivation of the Notch pathway by recruiting the Notch1 receptor to autophagosome-precursor vesicles and mediating its subsequent degradation [78]. Enhanced degradation of Notch1 or NICD via autophagy has also been observed in other cells types [72,73,79,80]. In contrast, our data suggest that autophagy supports Notch signaling. In our study, VEGF activated the Notch pathway in endothelial monolayers in a time-dependent manner, which may facilitate cell quiescence and junctional stabilization. Importantly, VEGF-induced NICD generation was significantly reduced in autophagy-depleted cells. Thus, the observed induction of autophagy by VEGF may be linked to NICD processing and ensure an appropriate adjustment of Notch signaling to the requirements of the complex angiogenic process. This will have to be substantiated in further experiments using three-dimensional models, which allow discrimination between tip and stalk cells, and monitoring the angiogenic process over time.

In summary, our data show that the growth factor VEGF is able to transiently stimulate autophagy via AMPK before it activates the mTOR pathway to induce cellular growth. Autophagy may have different functions in mediating angiogenesis including cellular homeostasis, the fast supply of macromolecules and processing of angiogenic molecules. One of these may be NICD, which regulates tip/stalk cell specification and vessel maturation. Our data highlight the importance of a well-balanced stimulation of AMPK and mTOR pathways in response to VEGF, which allows timely activation/inactivation of ULK1, a key player in autophagy initiation. As a consequence, temporary autophagy induction occurs and may promote angiogenic processes.
